# Intestinal UDP-glucuronosyltransferase as a potential target for the treatment and prevention of lymphatic filariasis

**DOI:** 10.1371/journal.pntd.0007687

**Published:** 2019-09-12

**Authors:** Alexander F. Flynn, M. Gordon Joyce, Rebekah T. Taylor, Sasisekhar Bennuru, Alyssa R. Lindrose, Spencer L. Sterling, C. Paul Morris, Thomas B. Nutman, Edward Mitre

**Affiliations:** 1 Department of Microbiology, F. Edward Hébert School of Medicine, Uniformed Services University of the Health Sciences, Bethesda, Maryland, United States of America; 2 U.S. Military HIV Research Program, Walter Reed Army Institute of Research, Silver Spring, Maryland, United States of America; 3 Henry M. Jackson Foundation for the Advancement of Military Medicine, Inc., Bethesda, Maryland, United States of America; 4 Department of Biology, Frostburg State University, Frostburg, Maryland, United States of America; 5 National Institute of Allergy and Infectious Diseases, National Institutes of Health, Bethesda, Maryland, United States of America; 6 Department of Pathology, Johns Hopkins Hospital, Baltimore Maryland, United States of America; University of Liverpool, UNITED KINGDOM

## Abstract

Lymphatic filariasis (LF), a morbid disease caused by the tissue-invasive nematodes *Wuchereria bancrofti*, *Brugia malayi*, and *Brugia timori*, affects millions of people worldwide. Global eradication efforts have significantly reduced worldwide prevalence, but complete elimination has been hampered by limitations of current anti-filarial drugs and the lack of a vaccine. The goal of this study was to evaluate *B*. *malayi* intestinal UDP-glucuronosyltransferase (Bm-UGT) as a potential therapeutic target. To evaluate whether Bm-UGT is essential for adult filarial worms, we inhibited its expression using siRNA. This resulted in a 75% knockdown of *Bm-ugt* mRNA for 6 days and almost complete suppression of detectable Bm-UGT by immunoblot. Reduction in Bm-UGT expression resulted in decreased worm motility for 6 days, 70% reduction in microfilaria release from adult worms, and significant reduction in adult worm metabolism as detected by MTT assays. Because prior allergic-sensitization to a filarial antigen would be a contraindication for its use as a vaccine candidate, we tested plasma from infected and endemic normal populations for Bm-UGT-specific IgE using a luciferase immunoprecipitation assay. All samples (n = 35) tested negative. We then tested two commercially available medicines known to be broad inhibitors of UGTs, sulfinpyrazone and probenecid, for *in vitro* activity against *B*. *malayi*. There were marked macrofilaricidal effects at concentrations achievable in humans and very little effect on microfilariae. In addition, we observed that probenecid and sulfinpyrazone exhibit a synergistic macrofilaricidal effect when used in combination with albendazole. The results of this study demonstrate that Bm-UGT is an essential protein for adult worm survival. Lack of prior IgE sensitization in infected and endemic populations suggest it may be a feasible vaccine candidate. The finding that sulfinpyrazone and probenecid have *in vitro* effects against adult *B*. *malayi* worms suggests that these medications have promise as potential macrofilaricides in humans.

## Author summary

*Brugia malayi* is a parasitic nematode and one of the causative agents of lymphatic filariasis, a disease that affects 70 million people worldwide. Currently, there are no effective therapeutics that kill adult filarial parasites when given as a short course. This limitation has hampered global eradication efforts. Studies have shown that the intestinal tract in nematodes can be effectively targeted by drugs and antibodies. Given this potential, we decided to investigate *B*. *malayi* intestinal UDP-glucuronosyltransferase as a potential therapeutic target. We determined that this protein is essential for *B*. *malayi* adult worm survival, as gene-expression knockdown rapidly decreased motility, fecundity, and microfilarial release. We also identified two FDA-approved UGT inhibitors that cause death of adult filariae *in vitro*. This is a critical finding due to the need for effective macrofilaricides and the potentially rapid translatability of these drugs for use in filaria-infected people. Finally, we showed that serum from filarial patients does not contain specific IgE to Bm-UGT and thus this protein would likely not induce allergic reaction if given as a vaccine antigen to endemic populations.

## Introduction

Lymphatic filariasis (LF) is a debilitating disease caused by the tissue-invasive nematodes *Wuchereria bancrofti*, *Brugia malayi*, and *Brugia timori*. Currently, there are ~ 70 million people infected worldwide and over a billion people at risk for infection [1]. Since 2000, the Global Programme to Eliminate Lymphatic Filariasis has substantially reduced the number of people infected or at risk for infection [1]. However, it has become apparent that new strategies must be implemented in order to attain global eradication of LF [2, 3].

Development of new therapeutics that target adult filarial worms would greatly enhance our ability to eliminate lymphatic filariasis. When given individually, the antifilarial drugs diethylcarbamazine (DEC), ivermectin (IVM), and albendazole are effective against the microfilaria (Mf) stage but exhibit little activity against adult filarial worms [4]. Use of all three medications together appears to have a macrofilaricidal effect [5], but due to the adverse effects caused by their potent microfilaricidal activity DEC and ivermectin cannot be used for mass drug administration (MDA) in areas endemic for *Loa loa* or *Onchocerca volvulus*. Therefore, development of a short-course macrofilaricidal agent or a vaccine would be very valuable for eradication efforts.

Unlike cestodes and trematodes, nematodes have a complete intestinal tract. Over the past 15 years, intestinal proteins of *Necator americanus* (hookworm) and *Haemonchus contortus* (barber pole worms) have been shown to be effective vaccine candidates in animal models [6–11]. Considering this work, our group performed a proteomic analysis of the intestine, body wall, and reproductive tract of adult *B*. *malayi* worms to potentially identify novel drug and vaccine targets for lymphatic filariasis [12]. We identified 396 proteins that were specific to the intestinal tract of the adult worms. Of these intestinal proteins, we selected a subset for evaluation as drug and vaccine candidates based on high homology with other filarial species, extracellular domains with accessibility to drugs and antibody, and predicted function.

In this study, an adult *B*. *malayi* intestinal protein, UDP-glucuronosyltransferase (Bm-UGT), was identified as a potential therapeutic target. The protein was predicted to have an enzymatic function that could be inhibited. Furthermore, structural analysis of Bm-UGT by InterPro revealed a large extracellular domain that could be targeted by therapeutics. We determined that this protein was essential for worm survival using small interfering RNA (siRNA) to knockdown expression. Importantly, we identified two FDA-approved commercially available UGT inhibitors that exhibit macrofilaricidal activity and display synergy with albendazole *in vitro*. Finally, we analyzed the antibody response against Bm-UGT in filarial patients and found that neither infected individuals nor endemic normals develop detectable levels of IgE against Bm-UGT, suggesting it would not induce allergic reactions if used in a vaccine.

## Results

### *B*. *malayi* intestinal UGT exhibits high homology to other filarial species

Previously, we reported that Bm-UGT (Bm17378) was a specific intestinal protein of *B*. *malayi* adult worms [12]. Sequence analyses indicated the presence of homologues in human filarial worms (*Brugia* sp., *W*. *bancrofti*, *L*. *loa)* with significant homology (>75% identity), and to a lesser extent (~35–40% identity) in other nematodes such as *Dirofilaria immitis*, *Haemonchus contortus*, *Ancylostoma* sp., *Strongyloides* sp., *Oesophagostomum dentatum* and *Toxocara canis*. The most similar human proteins were UDP-glucuronosyltransferases as expected, but with low sequence identity <27%. Given the high predicted homology of Bm-UGT between *B*. *malayi* and *D*. *immitis*, we also evaluated orthologs in cat and dogs. Results of the sequence analyses revealed little homology to Bm-UGT.

**Fig 1:**
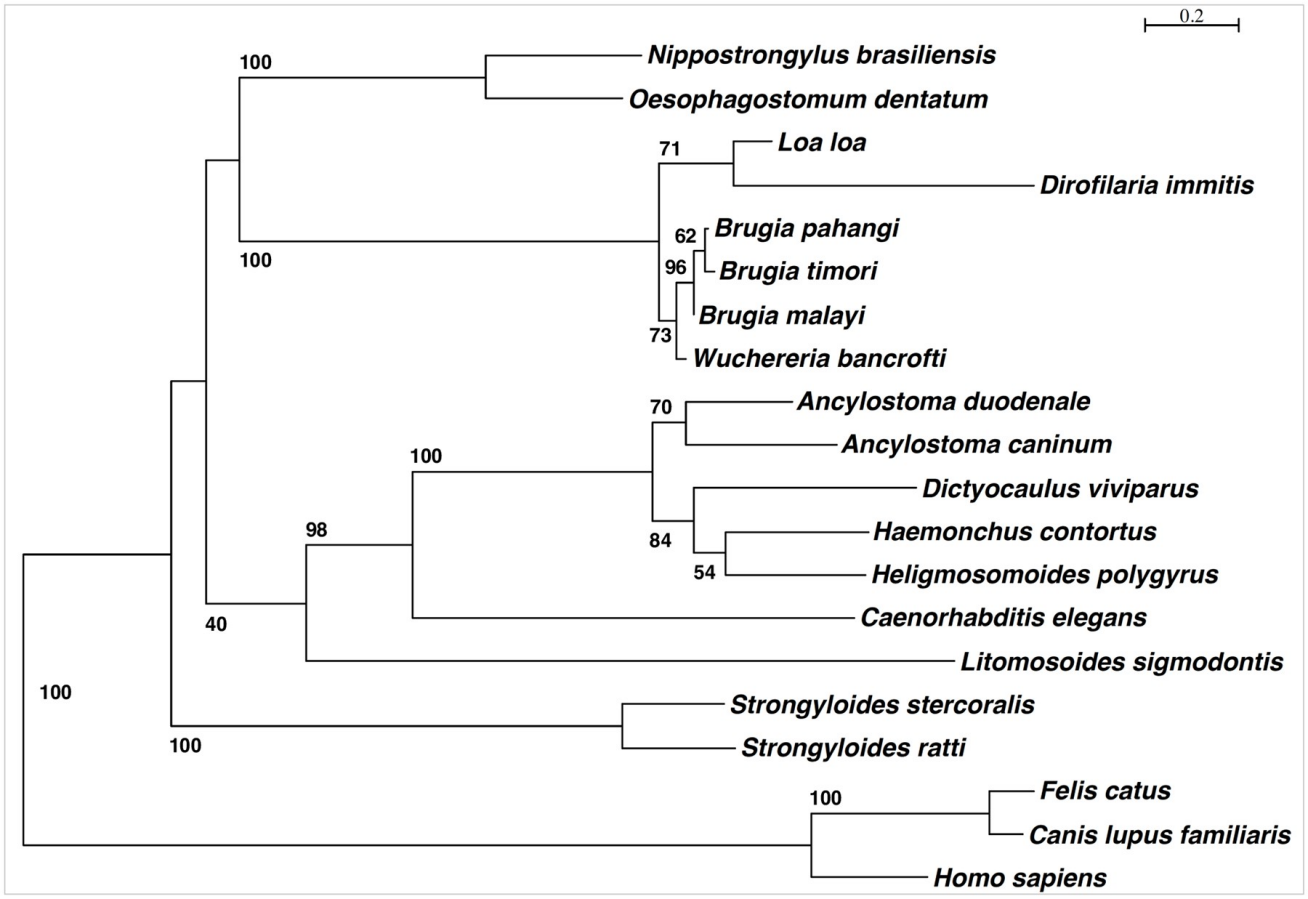
Phylogenetic comparison of Bm-UGT. Based on the *B*. *malayi* cDNA sequence alignment, there is a high level of relatedness to other filarial species for Bm-UGT and vast evolutionary distance to cat, dog, and human UGT molecules. The sequence alignment was generated using MUltiple Sequence Comparison by Log-Expectation (MUSCLE) performed by software SeaView. The tree was constructed based on the maximum likelihood method. The phylogenetic scale indicates genetic change as measured by the number of nucleotide substitutions per site.

We then generated a phylogenetic tree by first aligning the Bm-UGT cDNA-derived peptide sequences using MUltiple Sequence Comparison by Log-Expectation (MUSCLE) and then creating a tree based on efficient maximum-likelihood estimation method by the LG model. As seen in Fig 1, there is a high level of relatedness between Bm-UGT and several filarial orthologs, including other *Brugia* species, *W*. *bancrofti*, *L*. *loa*, and *D*. *immitis*. Interestingly, we could find no UGT ortholog in *O*. *volvulus*, and relatedness to the ortholog in *Litomosoides sigmodontis*, a common murine model of filarial infection, is low. Importantly, there is significant evolutionary distance between the Bm-UGT and orthologs in humans, cats, and dogs.

### *Brugia malayi* iUGT expression is stage specific

Evaluation of data available from prior transcriptomic and proteomic studies of *B*. *malayi* demonstrates that Bm-UGT is not expressed in all the lifecycle stages (Table 1). A study by Li et al. shows that Bm-UGT transcript is only expressed in third stage larvae (L3s) and adult female and male worms [13]. In addition, an RNAseq study by Choi et al. on various lifecycle stages of *B*. *malayi* found that Bm-UGT was preferentially expressed during later larval stages. Consistent with these findings, Bm-UGT protein expression was found to be specific to these stages as well [14].

**Table 1:** Stage specific expression of Bm-UGT. *Bm-ugt* transcript is expressed in both sexes of the *B*. *malayi* adult filaria as well as the L3 larval stage. Protein expression of Bm*-*UGT is consistent with this transcript profile.

	Adult Male	Adult Female	L3Larvae	MF	Immature MF
***Bm-ugt* transcript**	**+**	**+**	**+**	–	–
**Bm*-*UGT protein**	**+**	**+**	**+**	–	–

### Structural analysis of Bm-UGT

**Fig 2:**
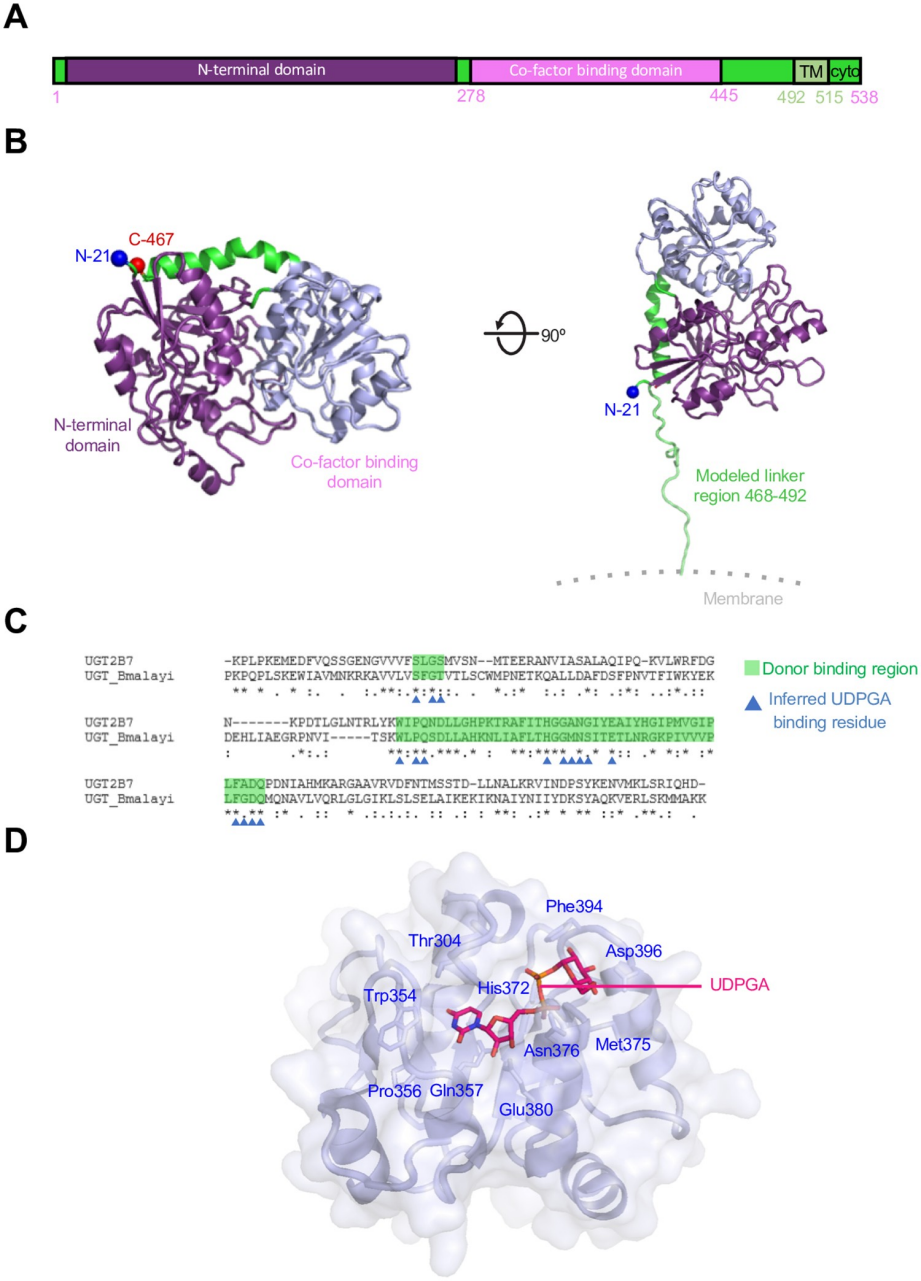
Structural analysis of filarial *B*. *malayi* intestinal UDP-glucuronosyltransferase (Bm*-*UGT). (A) Schematic representation of the Bm-UGT protein domains. (B) Structure model of the UDP-glucuronosyltransferase generated using SWISS-MODEL and PDB ID: 5NLM (UDP-glucosyltransferase from *Polygonum tinctorium*). The structure has a N-terminal domain (purple) and a co-factor binding domain (grey) with linker residues 468–492 modelled (right). (C) Sequence alignment of UGT2B7 C-terminal domain (PDB ID: 2O6L) with the filarial Bm-UGT C-terminal domain. Putative co-factor binding residues are indicated based on sequence and structural homology. (D) Structure of the co-factor binding domain is shown with transparent surface representation and inferred UDPGA-interacting residues shown in stick representation and labeled. The UDPGA location is modelled based on the plant glycosyltransferase *Medicago truncatula* UGT71G1—UDP-glucose structure (PDB ID: 2ACW).

Predictive analysis using the InterPro database revealed that the protein contains a large luminally-expressed domain likely accessible to small molecules or ingested antibodies. Sequence analysis of the Bm-UGT (Fig 2A) indicates that residues 1–20 encode a signal peptide [15] followed by a two-domain UGT (residues 21–278: N-terminal domain; 278–445: co-factor binding domain), a linker region, a transmembrane domain, and a short intra-cellular domain. The structure of the Bm-UGT was modeled using SWISS-MODEL with 43 template structures utilized. The final model was based on Protein Data Bank (PDB): 5NLM (the structure of the *Polygonum tinctorium* UGT) with a sequence identity of 19.7% [PMID: 29309053] (Fig 2B). UGTs add a glucuronic acid moiety to a substrate by transfer of the glucuronosyl group from uridine 5’-diphospho-glucuronic acid (UDPGA). Further structure-based searches using the PDB identified the 2B7 UGT as a model for the co-factor binding domain, and based on the co-factor interacting residues, the Bm-UGT has a potential UDPGA-binding site (Fig 2C and 2D). The enzyme nucleotide-sugar binding sites utilize a common structural scaffold; while specific interactions with the donor ligands vary between enzymes, analysis of the proposed binding site demonstrates it contains significant sequence homology to the active site of other UGTs.

### Uptake of target specific siRNA in the intestinal tract of adult *B*. *malayi* worms

**Fig 3:**
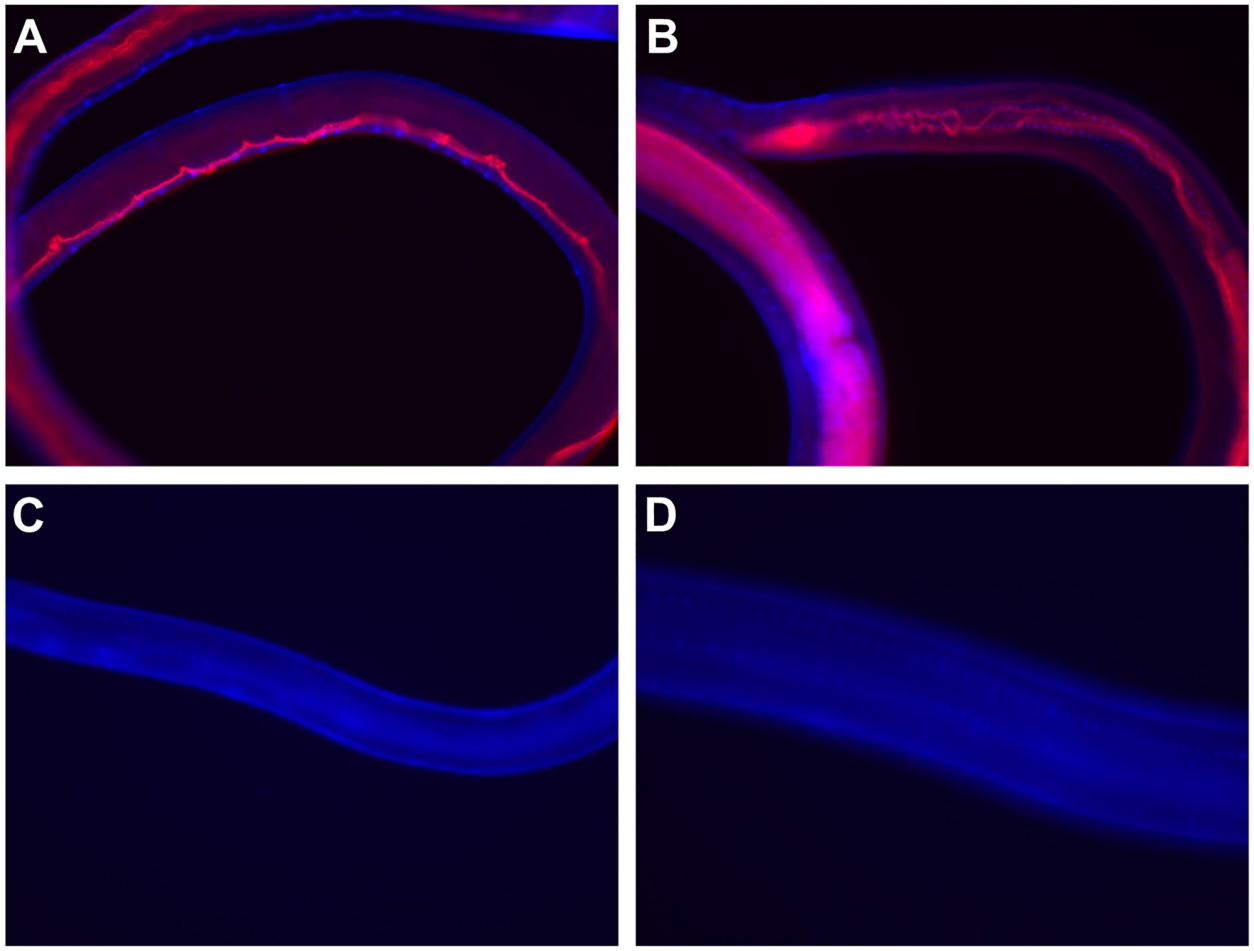
Cy3-labeled Bm-UGT siRNA enters the intestinal tracts of female *B malayi* adult worms. *B*. *malayi* adult worms incubated with Cy3-labeled siRNA (red) for 24 hrs and visualized at magnifications of (A) 100x and (B) 200x. Worms incubated in media alone for 24 hrs and visualized at magnifications of (C) 100x and (D) 200x. Worms were counterstained with DAPI (blue).

To evaluate whether siRNA is taken up by adult *B*. malayi worms, Cy3-conjugated Bm-UGT siRNA was added to the culture media of adult *B*. *malayi* worms for 24 hrs. Visualization of the adult worms shows clear uptake of siRNA throughout the intestinal tract (Fig 3A and 3B). In contrast, imaging at the same exposure time reveals no apparent signal in the intestine of adult *B*. *malayi* cultured in media alone (Fig 3C and 3D).

In addition, we also confirmed that antibodies could access the lumen of the intestine via ingestion by the adult filaria (S1). Adult filaria worms were incubated with Cy3-labeled mouse IgG for 24 hours and then viewed under a fluorescent microscope. The labeled antibodies emitted a positive signal in the gut of the adult female worms while no signal was detected in worms incubated in media alone.

### Bm-UGT siRNA reduces target transcript and protein expression

**Fig 4:**
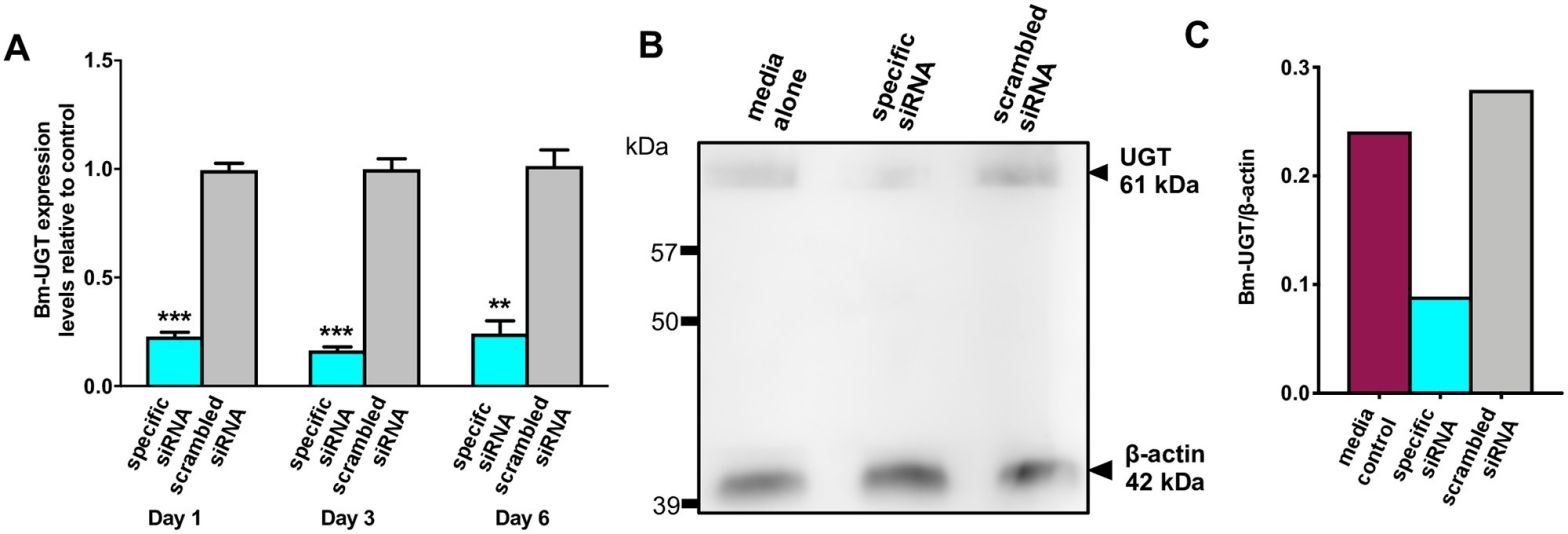
Bm-UGT siRNA incubation reduces target transcript and protein levels in adult *B*. *malayi* worms. Adult female worms were incubated with Bm-UGT siRNA, scrambled siRNA, or media alone. (A) Target mRNA levels (n = 3) in the specific siRNA and scrambled siRNA treated groups were measured by RT-qPCR relative to the media control normalized to *Bm-gapdh*, (Day 1, p = 0.0002; Day 3, p<0.0001; Day 6, p = 0.0034). p values were generated using an ordinary one-way ANOVA followed by Tukey’s multiple comparison test. These experiments were successfully repeated twice and the data shown is from a single representative experiment; mean ± SEM. (B) Bm-UGT expression (n = 5) was evaluated 24 hrs post-siRNA incubation using anti-Bm-UGT peptide antibodies. (C) Western blot quantification was performed using the ImageStudioLite software. Depicted are the signal intensities of anti-UGT normalized to beta-actin.

We next evaluated reduction in target transcript and protein levels. We selected timepoints of 1, 3, and 6 days post-siRNA treatment based on a protein half-life of approximately 10 hrs for UDP-glucuronosyltransferases (UGT) [16]. After a 24-hr incubation with target-specific siRNA or scrambled siRNA, we compared *Bm-ugt* mRNA expression between the treated worms relative to the media control by RT-qPCR (Fig 4A). Transcript expression was normalized employing the housekeeping gene *glyceraldehyde-3-phosphate dehydrogenase* (*Bma-gapdh*, *Bm5699*). Bm-UGT siRNA treatment resulted in a 77.1% reduction in target mRNA compared to the controls 1-day post-siRNA treatment (p = 0.0056). Transcript knockdown was sustained throughout the experiment with a 76.2% decrease in *Bm-ugt* transcription 6 days post-siRNA incubation (p = 0.0003 compared to controls). As expected, there was no significant difference in target transcription between the media control and scrambled siRNA groups. In addition, we did observe several worms (n = 3 at day 2 post-siRNA incubation) in the UGT siRNA group have no movement early into the experiment without any recovery throughout the course of the experiment.

Bm-UGT knockdown was further substantiated by immunoblotting (Fig 4B). Target protein expression was evaluated 24 hrs post-siRNA incubation using anti-Bm-UGT peptide antibodies. There was a robust reduction in UGT expression with the Bm-UGT siRNA treated worms compared to controls normalized to β-actin (68.1% reduction in Bm-UGT/β-actin, Fig 4C).

### Bm-UGT knockdown results in decreased worm viability and fecundity

**Fig 5:**
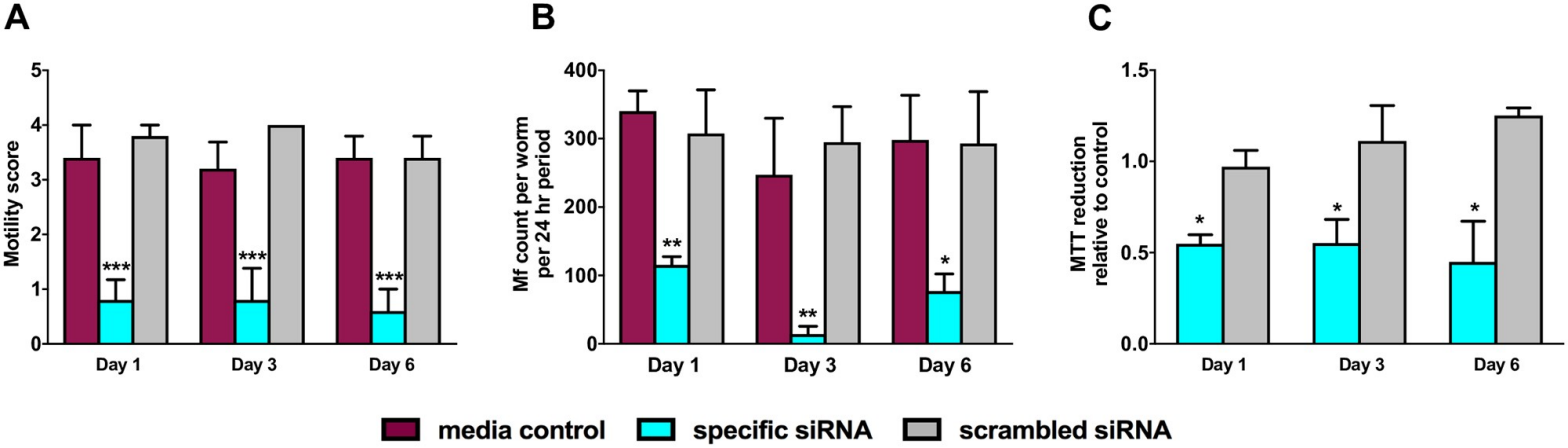
Bm-UGT expression knockdown decreases worm motility, microfilariae release, and metabolism. Adult female worms (n = 5) were incubated with Bm-UGT siRNA, scrambled siRNA, or media alone. Bm-UGT knockdown in female *B*. *malayi* adult worms caused reductions in (A) motility (n = 5; Day 1, p = 0.0006; Day 3, p = 0.0007; Day 6, p = 0.0004), (B) microfilaria count per worm (n = 5; Day 1, p = 0.0048; Day 3, p = 0.0096; Day 6, p = 0.0347) per 24-hr period, and (C) metabolism (n = 2; Day 1, p = 0.0148; Day 3, p = 0,0373; Day 6, p = 0.0243) as measured by MTT reduction relative to the media control at timepoints 1, 3, and 6 days post-siRNA treatment. For motility (A) and microfilaria release (B), p values were generated using an ordinary one-way ANOVA followed by Tukey’s multiple comparison test. For metabolism (C), p values were generated by an unpaired t-test. These experiments were successfully repeated twice and the data shown is from a single representative experiment; mean + SEM.

After successfully demonstrating that siRNA reduces Bm-UGT expression, we evaluated for any resultant changes in worm motility, Mf release, and metabolism. Adult worm motility was scored on a scale from 0 to 4, with 0 indicating no movement and 4 indicating active movement. At day 1 post-siRNA incubation, we observed a 77.1% reduction in motility with the Bm-UGT siRNA-treated group compared to the scrambled control (p = 0.0006, Fig 5A). This dramatic reduction in motility was maintained through day 6 (78.94% reduction, p = 0.0004).

We also observed a dramatic decrease in Mf release per adult worm per 24-hr period after Bm-UGT knockdown. At day 1, there was a 62.5% reduction from the specific siRNA-treated worms compared to the scrambled siRNA-treated group (p = .0048, Fig 5B). The greatest reduction in Mf release occurred at day 3 and was marked by a 95.3% difference between the Bm-UGT siRNA group and the scrambled control (mean number of Mf release in 24h = 14.0 vs 294.8, p = 0.0096).

Finally, we evaluated metabolism using a (4,5-dimethylthiazol-2-yl)-2,5-diphenyltetrazolium bromide (MTT) reduction assay. Decreased MTT reduction by the Bm-UGT siRNA treated *B*. *malayi* was observed at all three timepoints (Fig 5C). The values observed were similar over the course of the experiment with day 6 post-siRNA incubation showing the greatest difference between the specific and scrambled siRNA groups (64.2% lower values in the *Bm-UGT* treated group, p = 0.0243).

### Probenecid and sulfinpyrazone exhibit macrofilaricidal activity *in vitro*

The dramatic decrease observed in worm viability and fecundity after siRNA inhibition demonstrated that Bm-UGT is an essential protein for adult *B*. *malayi* survival. We next sought to evaluate the effects that UGT inhibitors had on adult worm survival. We tested two non-specific UGT inhibitors that act on multiple UGT isoforms for activity against *B*. *malayi* adult worms *in vitro*. Both of these agents, sulfinpyrazone and probenecid, are FDA-approved medications that are used to treat gout [17–20]. We incubated adult worms at various concentrations and found that both drugs killed filariae *in vitro* using worm motility as a metric for viability.

**Fig 6:**
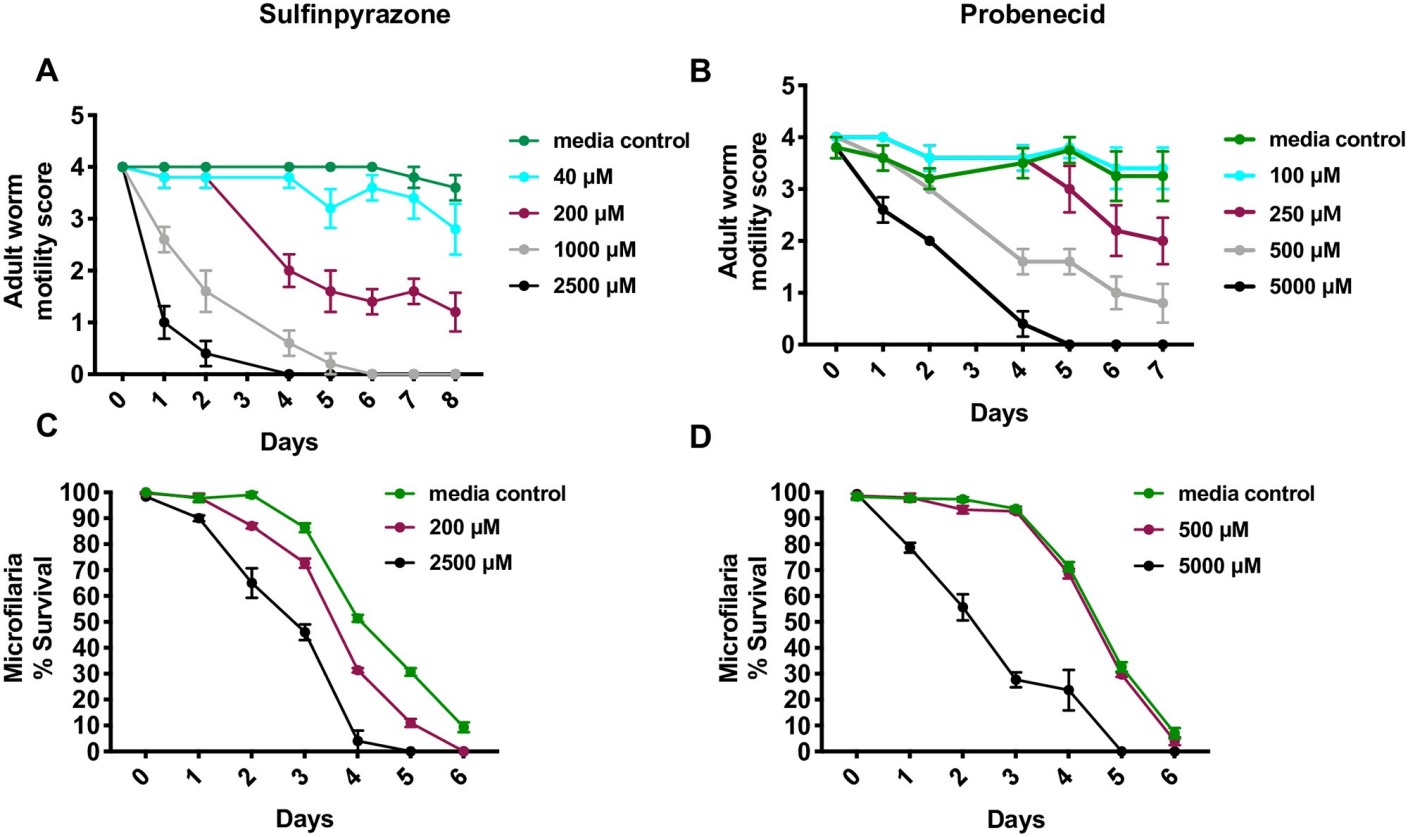
UGT inhibitors exhibit macrofilaricidal activity *in vitro*. Motility scores of adult *B*. *malayi* adult worms after incubation with various concentrations of (A) sulfinpyrazone (n = 5 for each group; 2500 μM, p<0.0001; 1000 μM, p<0.0001; 200 μM, p<0.0001; 100 μM, ns) and (B) probenecid (n = 5 for each group; 5000 μM, p<0.0001; 500 μM, p = 0.0004; 250 μM, ns; 40 μM, ns). For (A) and (B), the area under the curve (AUC) was calculated using the PRISM 7.0 software. Using these AUC values, a two-way ANOVA was performed to determine significance followed by Tukey’s multiple comparison test. Percent survival of *B*. *malayi* microfilariae incubated with (C) sulfinpyrazone (2500 μM, p<0.0001; 200 μM, p = 0.0005) and (D) probenecid (5000 μM, p<0.0001; 500 μM, ns). Percent survival was calculated from the first 100 Mf observed. The generated AUC values were analyzed using an ordinary one-way ANOVA to determine significance followed by Tukey’s multiple comparison test. These experiments were successfully repeated twice.

For sulfinpyrazone, we observed a dose-response relationship for macrofilaricidal activity *in vitro* (Fig 6A). The most rapid decline in adult worm motility occurred at 2500 μM. At this concentration, the area under the curve (AUC) was 3.6 and significantly different than the AUC of 31.6 for the control worms incubated with media alone (p<0.0001). Macrofilaricidal activity was also seen at 200 μM. The AUC at this concentration was 19.7 and significantly different (p<0.0001) than the AUC for media control. For probenecid, we also observed a dose-response curve for macrofilaricidal activity *in vitro* (Fig 6B). The greatest reduction in worm motility occurred at 5000 μM, which had an AUC of 8.1 (p<0.0001) compared to the media control AUC of 24.2. The lowest concentration that exhibited a significant effect on motility was 500 μM (p = 0.0004). Though worm motility at 250 μM was not significantly different over the course of the experiment, at day 7 there was a significant difference (p = 0.0105) between the treatment group compared to the control. For both UGT inhibitors, there were doses (>1000 μM sulfinpyrazone and 5000 μM for probenecid) that resulted in worms exhibiting no movement early in the experiment. Drug treatment was stopped for these worms and there was no recovery throughout the course of the experiment.

After testing the drugs on adult filariae, we tested for microfilaricidal effect. We observed modest microfilaricidal effects at the highest concentrations for both drugs (Fig 6C and 6D). However, no clear microfilaricidal effect was demonstrated for probenecid at 500 μM and very little for sulfinpyrazone at 200 μM.

While these drugs are FDA-approved, we wanted to determine whether the concentrations used were cytotoxic. Employing a lactate dehydrogenase (LDH) cytotoxicity assay with human embryonic kidney (HEK) cells, we did not detect any cytotoxicity at the concentrations that exhibited macrofilaricidal activity (S1 Table).

### UGT inhibitors and albendazole exhibit synergistic macrofilaricidal activity *in vitro*

After observing the macrofilaricidal effect of sulfinpyrazone and probenecid, we decided to investigate whether synergy existed between these inhibitors and albendazole. We hypothesized that because UGTs are involved in drug detoxification [21], inhibition of these enzymes may potentiate the effect of albendazole on filaria. In fact, one study showed that *C*. *elegans* treated with albendazole had upregulation of UGTs and metabolism of albendazole to albendazole-glucosides [22]. Because of this previous finding, we decided to incubate adult filaria with a sub-macrofilaricidal concentration of sulfinpyrazone (40 μM) or probenecid (100 μM) in combination with albendazole (10 μM).

**Fig 7:**
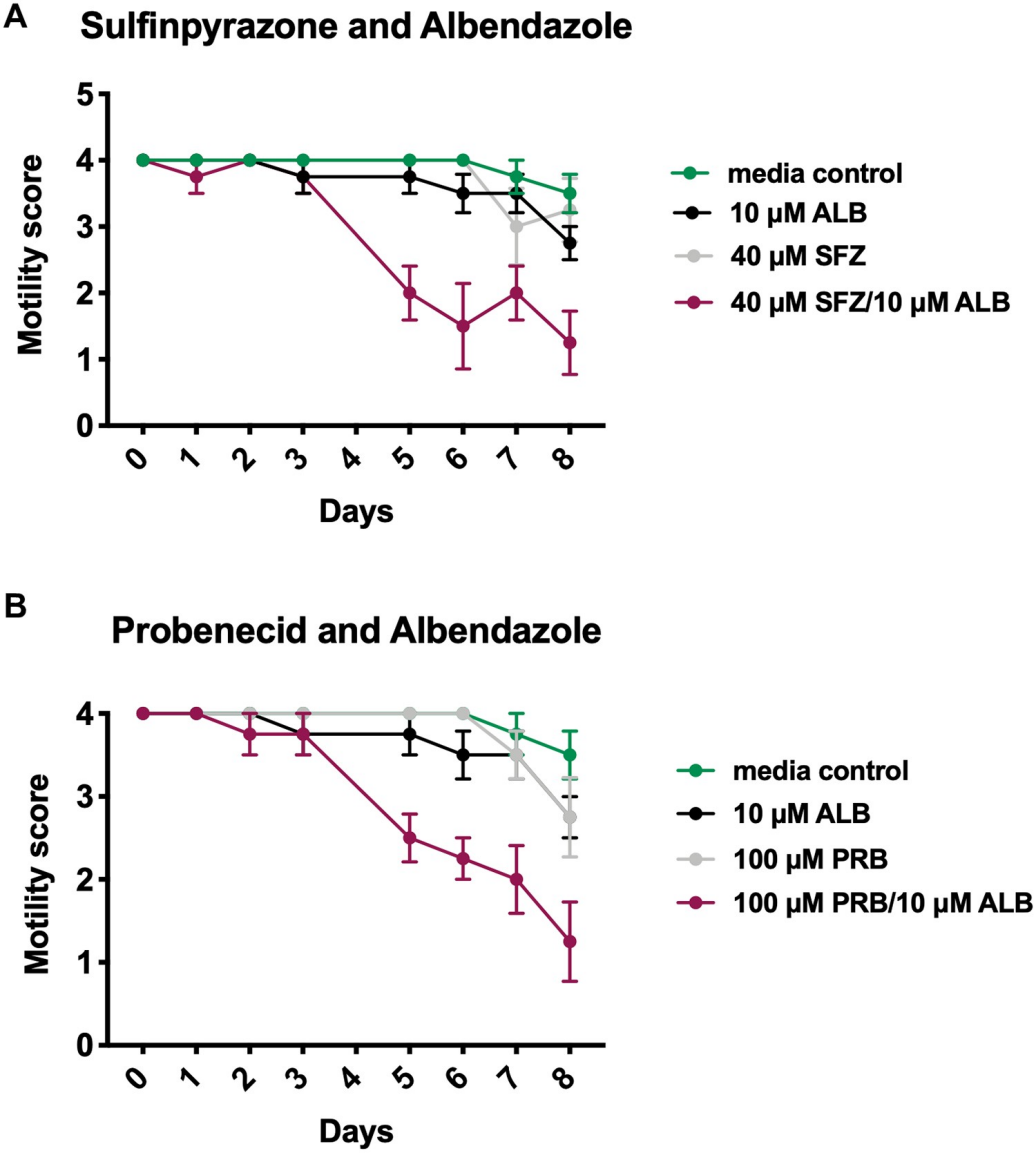
UGT inhibitors exhibit synergy with albendazole *in vitro*. Motility scores of adult *B*. *malayi* adult worms after incubation with 10 μM albendazole and (A) 40 μM sulfinpyrazone (n = 4, p = 0.0001) or (B) 100 μM probenecid (n = 4, p = 0.0022). For (A) and (B), the generated AUC values were analyzed by two-way ANOVA to determine significance followed by Tukey’s multiple comparison test. These experiments were successfully repeated three times.

Mild macrofilaricidal activity was observed with the 10 μM of albendazole treatment. Treatment of adult filariae with sulfinpyrazone in combination with albendazole (Fig 7A) produced an AUC of 22.5 based on worm motility. This was significantly lower than the resultant AUCs from treatment with sulfinpyrazone (30.63, p = 0.001) or albendazole (29.63, p = 0.0007). With the probenecid/albendazole combination (Fig 7B), we observed a significant decrease in worm motility resulting in an AUC of 24.0. This result was significantly different from a single treatment with either probenecid (AUC = 30.88, p = 0.0007) or albendazole (AUC = 29.63, p = 0.0022). Further, no synergistic microfilaricidal effects were observed with albendazole and sub-optimal concentrations of sulfinpyrazone (40μM) or probenecid (100 μM) (S2 Fig).

### Serum from patients infected with or exposed to lymphatic filariae does not contain IgG or IgE antibodies specific for Bm-UGT

A major obstacle for helminth vaccine development is the potential for individuals living in endemic countries to have pre-existing antigen-specific IgE and thus be at risk for developing allergic reactions when vaccinated with helminth antigens [23]. In this study we employed a luciferase immunoprecipitation system (LIPS) assay to determine whether individuals infected with filariae developed Bm-UGT-specific antibodies. Lysate containing Bm-UGT-luciferase fusion protein was incubated with serum from *W*. *bancrofti* infected individuals that were categorized as having asymptomatic microfilaremia (n = 13), chronic pathology (lymphedema) (n = 9), or tropical pulmonary eosinophilia (n = 8). All patients were untreated at the time of the blood draw. We also tested serum from individuals living in endemic areas that had no evidence of infection (endemic normal, n = 5). Healthy normal blood bank donor sera were used as negative controls (n = 5), while anti-Bm-UGT peptide antibodies raised in New Zealand rabbits served as positive controls. In addition, the naïve rabbit sera served as a negative control.

**Fig 8:**
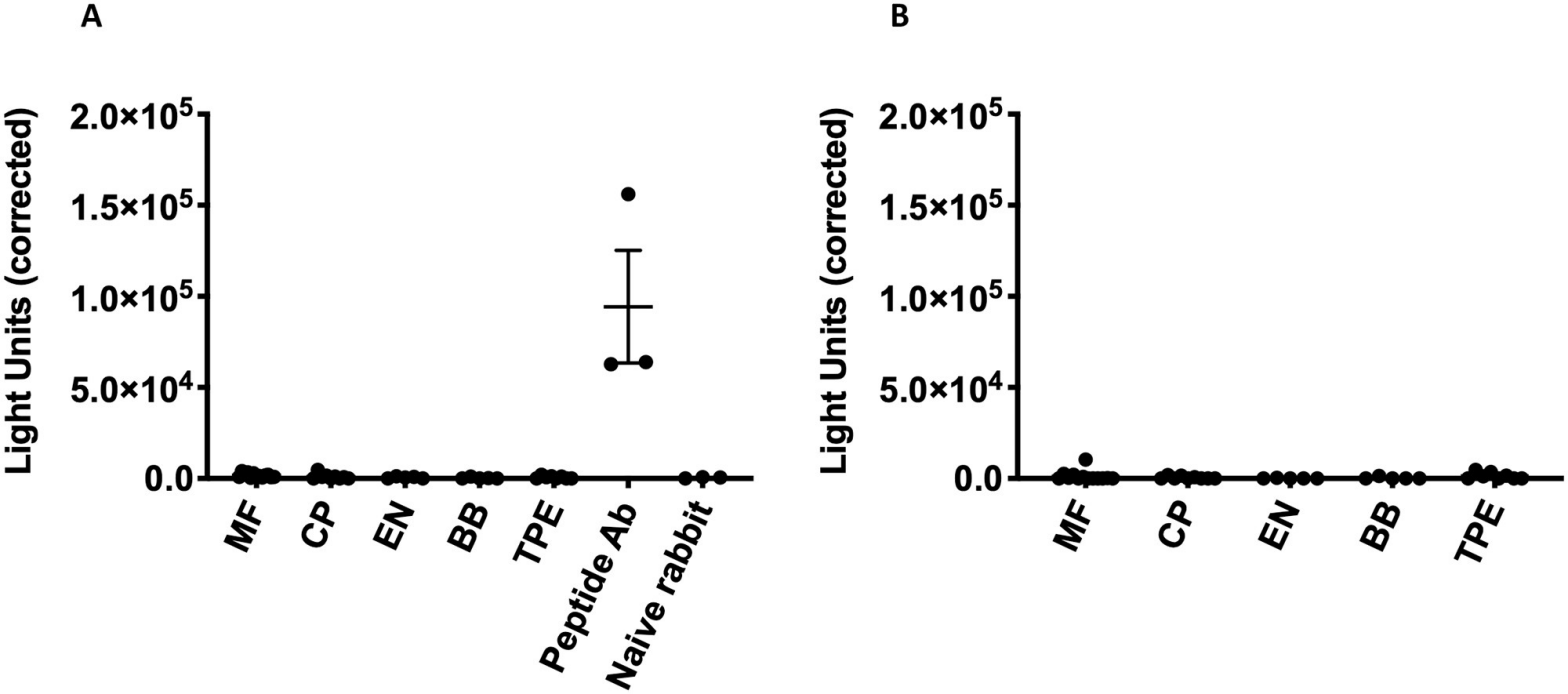
Serum of filaria-infected individuals does not contain detectable IgG or IgE against Bm-UGT. Filarial patient serum was incubated with a Bm-UGT-luciferase fusion protein. No detectable (A) IgG and (B) IgE was measured using the LIPS assay. The Bm-UGT peptide rabbit polyclonal antibodies served as a positive control for the detection of IgG. MF = microfilaremic, CP = chronic pathology, EN = endemic normal, BB = blood bank donors, TPE = tropical pulmonary eosinophilic, and Peptide Ab = Bm-UGT peptide polyclonal antibodies.

The LIPS assay did not detect Bm-UGT-specific IgG (Fig 8A) or IgE (Fig 8B) in any of the serum samples from filaria infected or exposed patients. As expected, there was no specific IgG or IgE in serum from U.S. blood bank donors while the Bm-UGT peptide IgG antibodies recognized our fusion protein.

## Methods

### Ethics statement

Infection studies conducted at FR3 and TRS were approved by their respective Animal Care and Use Committees, and protocols that enable receipt of filarial worms from FR3 and TRS for use at the Uniformed Services University of the Health Sciences (USUHS) were approved by the USUHS Animal Care and Use Committee. The generation of peptide antibodies by Genscript was on a protocol approved by the Genscript institutional animal care and use committee. Blood samples were obtained from patients and healthy volunteers who provided written consent under protocols approved by the NIAID’s Institutional Review board. All human subjects were adults.

### Parasites and culture

Female *B*. *malayi* adults used in this study were obtained from the NIH/NIAID Filariasis Research Reagent Resource Center (FR3) and TRS Laboratories in Athens, Georgia, USA. The worms were cultured in Dulbecco’s Modified Eagle’s Medium (Corning cellgro) supplemented with 10% heat-inactivated fetal bovine serum (Atlanta Biologicals), 100 units/mL of penicillin, 100 ug/mL of streptomycin, and 1% L-glutamine (Sigma) for 24 hrs at 37°C in 5% CO_2_ prior to siRNA treatment. Microfilariae were obtained from adult female worms cultured *in vitro*.

### Phylogenetic tree analysis

Orthologs in other nematode species were identified in WormBase Parasite based on a BLAST query [24] against the Bm-UGT protein sequence (Bm17378). The following are the accession numbers of each ortholog as identified in WormBase Parasite: *Brugia timori* (BTMF_0001026401), *Wuchereria bancrofti* (WBA_0000030501), *Brugia pahangi* (BPAG_0000208101), *Loa loa* (LOAG_03428), *Dirofilaria immitis* (nDi.2.2.2.t06727), *Litomosoides sigmodontis* (nLs.2.1.2.t00666-RA), *Ancylostoma caninum* (ANCCAN_05977), *Anyclostoma duodenale* (ANCDUO_14383), *Dictyocaulus viviparous* (NDV.1.0.1.g111112), *Haemonchus contortus* (HCON_00121250), *Heligmosomoides polygyrus* (HPOL_0001615101), *Nippostrongylus brasiliensis* (NBR_0001252501), *Caenorhabditis elegans* (Y37E11AR), *Strongyloides ratti* (SRAE_2000477000), *Strongyloides stercoralis* (SSTP_0001129400), and *Oesophagostomum dentatum* (OESDEN_03545).

Orthologs in selected mammals were identified in the National Center of Biotechnology Information (NCBI) databases based on a BLAST query against the Bm-UGT peptide sequence. The following are the orthologs selected for analyses: *Homo sapiens* (NP_066307), *Canis lupus familiaris* (XP_005635657), and *Felis catus* (BAA2492).

### Structural analysis of Bm-UGT

The Bm-UGT sequence was initially analyzed for properties including signal peptide sequence, and potential transmembrane sequence using InterPro, SignalP4.1 (PMID: 28451972) and TM servers [15]. Using the SWISS-MODEL homology modelling server [PMID: 29788355], the iUGT sequence was used to search against the SWISS-MODEL template library using BLAST and HHBlits for structures that matched the target sequence. The model was visualized using PyMOL [25] and COOT [PMID: 20383002], with residues 468–492 manually built using COOT. Based on sequence and structure identity, the co-factor binding domain was further analyzed, utilizing the inferred UDPGA-binding site of Bm-UGT mapped using the UGT 2B7 structure [PMID: 17442341] as a model and visualized using COOT [PMID: 20383002].

### siRNA design

Using the BLOCK-iT^™^ RNAi Designer, we selected the top three Bm-UGT siRNA duplexes for gene silencing activity and specificity. The Bm-UGT siRNA and corresponding scrambled siRNA were synthesized by Life Technologies and purified by standard desalting methods. The 5’-3’ sequences of the Bm-UGT siRNA strands were as follows:

Bm-UGT siRNA 1
  sense: 5’ GCCUAACGAAACUAAGCAAdTdT 3’
  antisense: 5’ UUGCUUAGUUUCGUUAGGCdTdT 3’

Bm-UGT siRNA 2
  sense: 5’ GGCUUCCACAAUCUGAUUUdTdT 3’
  antisense: 5’ AAAUCAGAUUGUGGAAGCCdTdT 3’

Bm-UGT siRNA 3
  sense: 5’ GGUGGUAUGAAUAGCAUAAdTdT 3’
  antisense: 5’ UUAUGCUAUUCAUACCACCdTdT 3’

### Demonstration of siRNA uptake and antibody ingestion by fluorescence microscopy

For demonstration of siRNA uptake, adult female worms were incubated in 5 μM of 5’ Cy3-labeled Bm-UGT siRNA 3 (Sigma Aldrich) for 24 hrs. Adult worms incubated in media alone were used as a negative control. Both groups of worms were then stained with 10 μg/mL of DAPI (Sigma-Aldrich) in PBS. Fluorescent images were captured by a Nikon Eclipse E600 fluorescent microscope and converged using NIS-Elements software. For experiments to test ingestion of antibody, adult female worms were incubated with 100 μg of mouse Cy3-labeled IgG isotype control in 2 mL of culture media. The worms were imaged 24 hrs later using the TRITC filter on a Zeiss Axio Observer.A1.

### siRNA incubation of *B*. *malayi* female adult worms

siRNA inhibition of Bm-UGT in *B*. *malayi* adult female worms followed a protocol established by Aboobaker et al. with minor modifications [26]. siRNA inhibition in filarial worms has well-known variability and difficulty [27, 28]. For this study, we analyzed data for experiments that received greater than 60% knockdown. For each timepoint, 5 adult female worms were soaked in an equal mixture of the Bm-UGT siRNAs at a final concentration of 5 μM in 850 μL of culture media in a 5000 MWCO Pur-A-Lyzer^™^ dialysis tube (Sigma-Aldrich). This concentration of siRNA was shown in multiple studies to be sufficient at silencing gene expression [26, 29–31]. The dialysis tubes were placed in 1 L beakers with 500 mL of culture media for 24 hrs at 37°C in 5% CO_2_. Similarly, 5 adult female worms were soaked in media alone or scrambled siRNA (5 μM) in dialysis tubes for each timepoint as experimental controls. After the 24-hr incubation, the worms for each group were carefully extracted from the dialysis tubes and individually placed into wells with 1 mL of media. The worms were evaluated at timepoints 1, 3, and 6 days post-incubation for transcript knockdown, worm motility, MTT reduction, and microfilariae release.

### Worm motility

Worms were visualized with a dissecting microscope by an observer blinded to treatment category. Motility of the adult female *B*. *malayi* worms was rated based on the following scale 4 = active movement, 3 = modest reduction in movement, 2 = severe reduction in movement, 1 = twitching, and 0 = no movement.

### MTT reduction

Metabolic function of the adult female worms was assessed by reduction of (4,5-dimethylthiazol-2-yl)-2,5-diphenyltetrazolium bromide (MTT) from Sigma using a protocol established by Comley et al [32]. For each group per timepoint, 2 worms were incubated in 0.5 mL of phosphate buffered solution (PBS) pH 7.4 with 0.5 mg/mL of MTT for 30 minutes at 37°C in 5% CO_2_. The worms were then transferred into separate wells of a 96-well plate containing 200 μL of DMSO and incubated at room temperature for 1 hr. MTT reduction was quantified by absorbance relative to a DMSO blank at 570 nm using a Synergy HTX multi-mode plate reader (BioTek).

### Microfilaria release

For each timepoint, adult worms were placed in new culture media 24 hrs prior to enumeration of microfilariae. After the overnight incubation, the worms were then removed for processing by the MTT reduction assay and RT-qPCR. The Mf in the well containing expended culture media (1 mL) were counted under a light microscope at high magnification.

### RNA extraction and analysis of RNA levels by RT-qPCR

For each condition, adult *B*. *malayi* female worms (n = 3) were homogenized in TRIzol (Thermo Fischer Scientific) after three freeze/thaw cycles using Matrix D lysis tubes (MP Biomedicals) agitated by a FastPrep^™^-24 Biopulverizer (MP Biomedicals) for 7 minutes at 6 m/s. Chloroform was added to the homogenate, transferred to Phase Lock Gel tubes (5Prime), and phase separated at 11,900 g for 15 minutes at 4°C. The aqueous phase was collected and cold isopropanol was added to precipitate the RNA, which was then pelleted at 12,000 g for 1 hr and washed twice using 75% ethanol. The RNA pellet was resuspended in nuclease-free water and quantified using a NanoDrop 1000 (Thermo Fischer Scientific). cDNA was prepared using Superscript IV (Thermo Fischer Scientific) as per the manufacturer’s protocol. The cDNA levels of Bm-UGT and *B*. *malayi* house-keeping gene *gapdh* were assessed in duplicate 20 uL reactions using 1 μL of 20X TaqMan^™^ gene expression assay (Thermo Fischer), 1 μL of cDNA, and 18 μL of TaqMan^™^ gene expression master mix (Applied Biosystems). PCR conditions were 2 min at 50°C, 10 min at 95°C, 40 cycles of 15 sec at 95°C, and 1 min at 60°C cycle of 50°C with a 7500 Real-Time PCR System (Applied Biosystems). The primers used were as follows:

*Bma-gapdh*:
  Forward primer: 5’ TTGATCTCACTTGCCGACTC 3’
  Reverse primer: 5’ TGGTCTTCGGTGTATTCCAA 3’
  Internal probe: 5’ CAGCTAATGGACCGATGAAGGGGA 3’

*Bm-ugt*:
  Forward primer: 5' TATCATTCGGCACCGTTACA 3'
  Reverse primer: 5' ATTCATACCACCATGCGTCA 3'
  Internal probe: 5' TCGCTGAGGGACGTCCAAACG 3'

### Generation of rabbit polyclonal antibodies against Bm-UGT peptides

Polyclonal anti-Bm-UGT peptide antibodies were generated in New Zealand rabbits by Genscript using Bm-UGT peptide sequences conjugated to keyhole limpet hemocyanin (KLH). The peptide sequences used were as follows: CYEKDEHLIAEGRPN, DSTGSKLAKTVKIDC, and CGQIANFDPYGRKMS. Cysteines were added at either the N- or C-terminus to facilitate KLH conjugation.

### Immunoblotting

*B*. *malayi* adult worms (n = 5) were incubated in 5 μM combination of Bm-UGT siRNA for 24 hrs using the previously mentioned method and transferred into individual wells with 1 mL of media. The adult worms were cultured for an additional 24 hrs and then homogenized in PBS (pH 7.4) and 4 μL Halt^™^ Protease Inhibitor Cocktail (Thermo Scientific) using Matrix D lysis tubes (MP Biomedicals) agitated by a FastPrep^™^-24 Biopulverizer (MP Biomedicals) for 3 minutes at 4 m/s. Protein levels were quantified by the Bradford protein assay (Bio-Rad). For immunoblot analysis, 10 μg of protein was separated on 10% Bis-Tris NuPAGE gel (Invitrogen) and blotted onto 0.2 μm nitrocellulose filter paper (Bio-Rad). After blocking overnight in 5% bovine serum albumin (BSA) in tris-buffered saline with 0.1% Tween 20 (TBS-T), the membrane was incubated with 1:4000 anti-UGT peptide antibodies (Genscript) and 1:1000 rabbit anti-β actin antibodies (Abcam) for 1 hr. Following this, the filter paper was washed with TBS-T and then incubated with 1:2000 horseradish peroxidase conjugated goat anti-rabbit IgG for 1 hr. The membrane subsequently washed and incubated in Chemiluminescent reagent, SuperSignal^™^ West Pico PLUS (Thermo Scientific), to visual the bands.

### Adult *B*. *malayi* worm incubation with probenecid and sulfinpyrazone

Sulfinpyrazone (ChemCruz) and probenecid (Invitrogen, water soluble formulation), broadly acting UGT inhibitors, were evaluated for macrofilaricidal activity *in vitro*. Sulfinpyrazone was resuspended in 1X PBS (pH 7.4) and 1% dimethylsulfoxide (DMSO, v/v) while probenecid was resuspended in deionized water. When testing sulfinpyrazone, adult *B*. *malayi* female worms were incubated in culture media with the drug for 8 days at concentrations of 2500 μM, 1000 μM, 200 μM, 40 μM, and 8 μM. For probenecid, adult female worms were incubated in culture media with the drug for 7 days at concentrations of 5000 μM, 500 μM, 250 μM, and 100 μM. Worms were transferred into new media with corresponding drug concentrations every day except day 4. As a negative control, worms were incubated in culture media alone with a similar volume of vehicle. Worm motility was scored using the previously mentioned scale for the course of the experiment. Worms that were scored a zero stopped receiving UGT inhibitor treatment.

For the albendazole synergy experiments, adult filariae were incubated in culture media with 40 μM of sulfinpyrazone or 100 μM of probenecid in combination with 10 μM of albendazole, which was resuspended in 1X PBS (pH 7.4) and 1% DMSO (v/v). The worms were scored for motility for 8 days and were transferred into new media with corresponding drug concentrations every day except day 4.

### *B*. *malayi* microfilariae incubation with UGT inhibitors

The above UGT inhibitors were evaluated for microfilaricidal activity *in vitro*. For each drug, experiments were performed in triplicate at a concentration of 2 x 10^4^ Mf/mL in culture media. Viability was determined by quantifying the number of motile larvae from 100 randomly selected Mf per well. The concentrations used for sulfinpyrazone were 2500 μM and 200 μM while the concentrations used for probenecid were 5000 μM and 500 μM. As a negative control, larvae were incubated in culture media alone.

### UGT inhibitor Cytotoxicity Assay

We measured cytotoxicity of the UGT inhibitors using a Pierce LDH Cytotoxicity Assay Kit (Thermo Scientific). HEK cells were seeded at 5 x 10^4^ per well in DMEM (Quality Biological) with 10% Hyclone Cosmic Calf Serum (Thermo Fischer), 200 μM of L-glutamine (Quality Biological), and 50 μg/mL of gentamicin (Quality Biological) at 37°C in 5% CO_2_. We then incubated the cells with various concentrations of the UGT inhibitors overnight. Following this, we transferred 50 μL of media from each well to a new 96-well plate and then added 50 μL of reaction buffer. We incubated the mixture for 30 minutes and then added 50 μL of stop solution. We measured absorbance at 490 nm and 680 nm. We employed the following controls: a spontaneous LDH activity control which was incubated with the vehicle only and a maximum LDH activity control which was incubated with nothing but later lysed prior to incubation with the reaction buffer. We calculated absorbance for each well by subtracting the 680 nm absorbance value (background) from the 490 nm absorbance value. We then calculated percent cytotoxicity using the following equation:

$$\%\text{Cytotoxicity}=\frac{\left(\text{UGT}\;\text{inhibitor}\;\text{LDH}\;\text{activity}-\text{Spontaneous}\;\text{LDH}\;\text{actvity}\right)}{\left(\text{Maximum}\;\text{LDH}\;\text{activity}-\text{Spontaneous}\;\text{LDH}\;\text{activity}\right)}$$

### Generation of Ruc-antigen fusion proteins

Bm-UGT was expressed as a *Renilla reniformis* luciferase (Ruc) fusion protein by cloning the *Bm-UGT* coding sequence in pREN2 (Genscript). The Bm-UGT signal sequence as predicted by signalP was removed prior to synthesis. Plasmid encoding the fusion protein was used to transformed TOP10 cells (Thermo Fischer) and plasmid DNA was obtained from colonies selected on kanamycin (50 μg/ml) as per the manufacturer’s guidelines (Qiagen Midi-Prep). 293F cells grown in 293 Freestyle Medium as suspension cultures were transfected with 30 μg of Bm-UGT-Ruc plasmid, at a final concentration of 1 μg per 1 x 10^6^ cells (Thermo Fischer Sceintific) per mL, and cultured at 37°C with 8%CO_2_ on a rotary shaker at 125 rpm. After 72hrs, the cells were pelleted and sonicated in LIPS lysis buffer (20 mM Tris pH 7.5, 100 mM NaCl, 5 mM MgCl_2_, 1% TritonX-100, 50% glycerol, protease inhibitors (Mini from Roche)). The lysate was centrifuged to remove cellular debris and supernatant containing the *Bm-UGT*-Ruc fusion proteins were stored at -80°C for later use.

### Luciferase immunoprecipitation system

Antibody titers were measured using a luciferase immunoprecipitation system (LIPS) assay [33–35]. For IgG and IgE quantification, serum was diluted to 1:100 and 1:10 respectively in 50 μL of LIPS master mix (20 mM Tris pH 7.5, 100 mM NaCl, 5mM MgCl_2_, 1% Triton X-100) and mixed with 50 μL of the UGT-Ruc fusion containing 1 x 10^6^ light units (LU) of protein in PBST (PBS with 0.05% Tween-20). The reaction mixture was incubated in a 96-well polypropylene plate for 10 minutes at room temperature and transferred to a 96-well high throughput screening filter plate (Milipore) containing 5 μL of a 50% suspension of Ultralink protein A/G (Pierce) or Ultralink anti-human IgE beads in PBS and incubated for an additional 15 minutes at room temperature. The plates were washed under vacuum with 200 μL of LIPS master 3x followed by PBS once. The relative light units (RLU) were measured with a Berthold LB 960 Centro microplate luminometer with 50 μL of coelenterzine solution (Promega). For these experiments, samples were run in duplicate, and the calculated RLU was adjusted for the measured RLU of UGT fusion protein without serum.

### Serum samples

All human serum samples were obtained following written informed consent from all subjects using Institutional Review Board-approved protocols that have been registered (NCT00001345, NCT00090662, NCT00342576). Patients were grouped into clinical categories as previously detailed [36].

### Statistical analysis

The siRNA and UGT inhibitor experiments were repeated two times under similar conditions. Data shown is from a single representative experiment. For the siRNA experiments, data was analyzed using one-way analysis of variance (ANOVA) or T-test by PRISM 7.0. Following ANOVA, individual comparisons of mean values were performed using Tukey’s multiple comparisons test. For the UGT inhibitor experiments, we performed AUC analysis followed by one-way ANOVA to determine significance. Statistical significance between the experimental and control groups was designated as follows: * for p values <0.05, ** for p values <0.01, and *** for p values <0.001.

## Discussion

UDP-glucuronosyltransferases (UGT) are enzymes important for detoxification of xenobiotics and homeostasis of endogenous molecules [21]. Specifically, these phase II enzymes increase the solubility of hydrophobic molecules by attaching sugar moieties such as glucuronic acid. Since this sugar molecule is negatively charged at physiological pH, anion efflux pumps are able to transport these molecules outside the cell [37]. In *C*. *elegans*, studies demonstrated that RNAi of detoxification enzymes results in lethality, sluggish movement, or impaired growth [38–40]. In addition, one study showed that glycosylation by phase II enzymes was important for the detoxification of albendazole in *C*. *elegans* [22]. While UGTs in helminths have not been studied extensively, there is evidence from intestinal helminths to suggest that these enzymes play a critical role in drug resistance [41–43].

In this study, we showed that *B*. *malayi* intestinal UGT expression could be silenced by specific siRNA. This knockdown caused a significant reduction in worm motility, fecundity, and metabolism. While these metrics alone do not determine worm survival, we are fairly confident these are appropriate surrogates. We observed several worms with a scored motility of zero early into the siRNA experiments that did not show any recovery over the course of the experiment. Thus, Bm-UGT appears essential for adult *B*. *malayi* worm survival. It is possible that the observed phenotypic changes occurred due to an imbalance in endogenous molecules. Past studies in mice and rats demonstrated that UGTs were critical for protection against free radicals, which if left unchecked could mediate damage to DNA, lipid membranes, and amino acids [44–47]. The reduction in microfilaria release was most likely due to decreased adult worm viability because Bm-UGT is not expressed in the Mf stage [13, 14]. We suspect that targeting Bm-UGT *in vivo* would leave the worms not only susceptible to endogenous free radicals but also to those released by host immune cells.

Interestingly, it should be noted that the *Brugia* intestinal UGT was predicted by InterPro to be localized to the plasma membrane, which would be a novel location for this family of enzymes that are typically found in the endoplasmic reticulum. The prediction software also determined that this protein has a large extracellular domain, which potentially makes it readily accessible to drugs or antibodies.

Development of short-course macrofilaricidal agents would greatly enhance LF eradication efforts. Because the aim of this study was to evaluate Bm-UGT as a potential drug and vaccine target, we investigated the effects of non-specific UGT inhibitors on adult filariae. We found two UGT inhibitors that are also FDA-approved to treat gout [17–20]. Both drugs, sulfinpyrazone and probenecid, exhibited macrofilaricidal activity *in vitro*. For worms that were scored a zero for motility, we did not see any recovery after cessation of the UGT inhibitor treatment. The lowest effective concentration for sulfinpyrazone was 200 μM. A previous study investigating sulfinpyrazone showed a maximum concentration (Cmax) in humans of 79.9 μM for a 400 mg dose [48]. Given that the daily maximum recommended dose for humans is 800 mg, we believe a Cmax similar to our lowest effective sulfinpyrazone concentration may be achievable in humans [49]. Therefore, we speculate that this drug could serve as a novel therapeutic against adult filariae. Likewise, we believe that the same applies to probenecid, which demonstrated robust macrofilaricidal activity *in vitro* at 500 μM. We also observed a significant reduction in motility by day 7 at 250 μM suggesting that this concentration may be effective at killing adult worms if given over a longer time course. In the context of physiological relevance, one pharmacokinetic study showed the peak concentration in humans given a single 2 g oral dose of probenecid to be 148.6 μg/mL (520.7 μM) with minimal adverse events [50]. Based on our *in vitro* data, this level could rapidly kill adult filarial worms.

While the UGT inhibitors displayed macrofilaricidal activity, it is possible that probenecid and sulfinpyrazone may act on adult *B*. *malayi* worms through mechanisms independent of effects on Bm-UGT. In addition to inhibiting UGTs, probenecid and sulfinpyrazone also inhibit organic anion transporters (OATs) [51, 52] and pannexins [53]. OATs function to transport negatively charged molecules and are likely important for adult filarial worm survival. Innexins, which are present in invertebrate organisms, are structurally very similar to pannexins and primarily function as membrane channels that communicate with the extracellular space [54]. Interestingly, probenecid has been shown to impair touch responses in *C*. *elegans* by inhibiting mechanosensitive innexin channels [55]. Elucidating the mechanisms by which probenecid and sulfinpyrazone kill adult filarial worms will be the focus of future studies.

While we are interested in developing a drug that kills adult filarial worms, the absence of microfilaricidal activity also presents advantages. Current antifilarial therapeutics such as diethylcarbamazine (DEC) and ivermectin (IVM) are extremely effective at clearing microfilariae. However, their use is contraindicated in areas co-endemic for loiasis and onchocerciasis because rapid killing of microfilariae in these infections can lead to severe adverse outcomes [56]. Individuals with loiasis when treated with IVM or DEC have a significantly higher risk of experiencing severe neurologic events such as encephalopathy due to rapid Mf death in the vasculature [57, 58]. Similarly, DEC can induce adverse systemic reactions such as skins lesions, fever, polyarthritis, and ocular reactions in patients with onchocerciasis as determined by Mf load [59, 60]. Therefore, probenecid, which demonstrated macrofilaricidal but not microfilaricidal activity at 500 μM *in vitro*, may be an attractive LF treatment candidate in these co-endemic areas.

Because UGTs are involved in drug metabolism, we suspected that there may be synergy between the UGT inhibitors and albendazole. A recent study demonstrated that overexpression of UGT-22 in *C*. *elegans* resulted in albendazole resistance [61]. This, coupled with an earlier study by Laing et al showing that upregulation of UGTs is associated with metabolism of albendazole into various glucuronide products [22], suggests that UGTs may play a critical role in nematode metabolism of albendazole. In support of this hypothesis, our data demonstrate a synergistic effect of sub-macrofilaricidal concentrations with sulfinpyrazone or probenecid in combination with albendazole against adult filaria *in vitro*. These results suggest that combination therapy with albendazole and either probenecid or sulfinpyrazone may be highly effective in treating filarial infections in people. Future studies will determine whether Bm-UGT functions to metabolize albendazole in filarial worms and whether this metabolism is inhibited by UGT inhibitors. We also plan to test the efficacy of combination therapy in animal models of infection.

As previously mentioned, these probenecid and sulfinpyrazone inhibitors are FDA-approved and have been shown to be safe in humans [62], with probenecid characterized as a pregnancy category B drug. If these medicines demonstrate macrofilaricidal activity *in vivo*, translation into human use could occur quickly.

In addition to being a potential drug target for filariasis, we postulate that Bm-UGT could serve as a vaccine candidate as well. One of the challenges in helminth vaccine development is the risk that the vaccine may induce an allergic response in endemic populations. Indeed, generalized urticaria was seen in several Brazilian patients immunized against *Ancylostoma*-secreted protein 2 during hookworm vaccine trials [23]. A solution to this obstacle is to identify “hidden antigens” which are not exposed to the immune system during natural infection yet are essential to worm survival and present in an anatomical location accessible to host antibodies [63, 64]. In theory, these proteins would not elicit an IgE-mediated response, and, as postulated by Munn, these antigens may be especially vulnerable to the immune system due to a lack of evolutionary pressure to evade it [64]. There is evidence to support that the intestinal tract of nematodes contains hidden antigens. Studies have demonstrated the absence of pre-existing IgE in serum from endemic populations against hookworm intestinal antigens APR-1 and GST [8, 9, 65]. Furthermore, studies have shown that these antigens are protective in animal models [8, 10, 11]. There is also evidence of hidden antigens in *H*. *contortus* seen with the lack of an antibody response against H11, a glycosylated intestinal protein [66]. In this study, we observed no detectable Bm-UGT-specific IgE in the serum from individuals infected with or exposed to lymphatic filariae, which suggests this antigen may be safe to administer as a vaccine candidate in endemic populations. However, due to the low numbers of patient samples tested, if vaccine work using Bm-UGT progresses, then future studies would need to evaluate potential for allergic responses by testing far larger numbers of individuals.

After demonstrating that recombinant Bm-UGT would not induce an allergic response in endemic populations, we investigated whether adult *B*. *malayi* worms could ingest antibody as there is a degree of uncertainty about whether filarial worms use their intestine to feed. Only the adult and L4 stages of filariae have a fully formed intestinal tract, while the Mf, L2, and L3 stages have an immature intestine inaccessible to nutrients [67]. Furthermore, past studies have already shown that *Brugia* worms are able to absorb nutrients such as nucleotides, amino acids, sugars, and vitamins through their cuticle, which calls into question the purpose of the intestinal tract [68]. Notwithstanding these findings, Attout et al. investigated the intestinal tract of *L*. *sigmodontis* and demonstrated that young adult worms (25–56 weeks post-infection) ingested red blood cells [69]. This suggests that intestinal feeding may occur but only at the early adult stage. Another study showed that *D*. *immitis* can ingest labeled serum [70]. With our study, we were able to show that adult *B*. *malayi* worms can ingest Cy3-labeled IgG (S1 Fig). This serves as clear evidence that circulating antibody can potentially access the intestine of adult filarial worms. However, not all the worms ingested the labeled antibody, which indicates that, at least during *in vitro* conditions, adult worms do not feed through the intestine continuously. Future studies will work towards recombinant expression of Bm-UGT in order to test its ability to induce protective immune responses in animal models of filariasis.

On the basis of the phylogenetics analyses we conducted, we expect that Bm-UGT may also be essential in *W*. *bancrofti* and *B*. *timori* given the overall high homology shared between these species and *B*. *malayi*. Additionally, there is a high level of sequence homology (> 70%) between the *B*. *malayi* intestinal UGT and the orthologs found in *D*. *immitis* and *L*. *loa* and thus the UGT orthologs in these filaria species may also serve as novel therapeutic targets. Interestingly, we did not find an ortholog of Bm-UGT in *O*. *volvulus* and speculate this could be the result of evolutionary pressure to lose this gene.

In summary, we believe that Bm-UGT is an essential intestinal protein in *B*. *malayi* adult worms that does not induce IgE antibodies in endemic populations. Importantly, we found that sulfinpyrazone and probenecid, two commercially available, FDA-approved medications in use for gout, exhibit strong macrofilaricidal activity *in vitro* at concentrations that are achievable in humans. This promising data warrants future investigation in animal models of *Brugia* infection as well as assessment of whether these UGT inhibitors exhibit macrofilaricidal activity *in vitro* against other filarial species. Furthermore, we demonstrated that probenecid and sulfinpyrazone may potentiate the effect of albendazole on filariae, suggesting that combination therapy may be an ideal approach to obtain macrofilaricidal effect. In terms of vaccine development, future studies will focus on recombinantly expressing Bm-UGT and then testing whether it is protective in animal models of filariasis. Finally, the results of this study suggest that the intestinal tract of filarial nematodes may serve as a rich source of essential proteins that can serve as important therapeutic targets.

## Supporting information

S1 Table LDH Cytotoxicity Assay for the UGT inhibitors. 2 x 10^4^ HEK cells were seeded in various concentrations of sulfinpyrazone and probenecid. LDH activity was measured by absorbance at 490 nm and 680 nm and was calculated by subtracting LDH activity at 680 nm from activity at 490 nm. Percent cytotoxicity was calculated against the maximum LDH activity (lysed cells) using spontaneous LDH activity (vehicle only) as a background control.
(TIF)

S1 Fig Adult *B*. *malayi* ingest mouse polyclonal IgG. Adult worms were cultured for 24 hrs with Cy3-labeled mouse polyclonal IgG or in media alone. Ingested antibody is visible in the intestinal of the adult filaria.
(TIF)

S2 Fig UGT inhibitors do not exhibit synergy with albendazole against B. malayi microfilariae. Percent survival of microfilariae incubated with 10 μM albendazole and 40 μM sulfinpyrazone (ns) and 100 μM probenecid (ns). Percent survival was calculated wells containing 2.0 x 10^6^ Mf similar to Fig 6. The generated AUC values were analyzed using an ordinary one-way ANOVA to determine significance followed by Tukey’s multiple comparison test. This experiment was only performed once.
(TIF)
