# Supplementary figures and images for: Intestinal UDP-glucuronosyltransferase as a potential target for the treatment and prevention of lymphatic filariasis
Source: PLoS Negl Trop Dis. 2019 Sep 12;13(9):e0007687. doi: 10.1371/journal.pntd.0007687 (PMC6742224; doi:10.1371/journal.pntd.0007687)

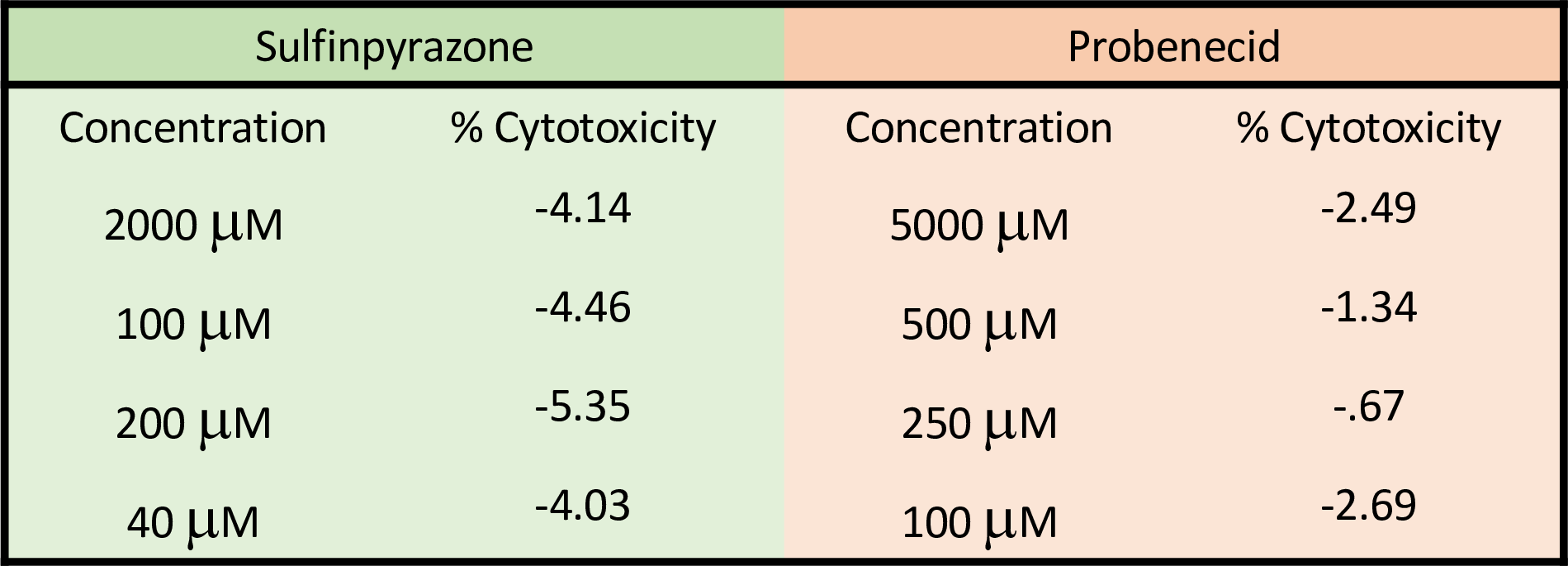

Supplement: S1 Table — 2 x 10^4^ HEK cells were seeded in various concentrations of sulfinpyrazone and probenecid. LDH activity was measured by absorbance at 490 nm and 680 nm and was calculated by subtracting LDH activity at 680 nm from activity at 490 nm. Percent cytotoxicity was calculated against the maximum LDH activity (lysed cells) using spontaneous LDH activity (vehicle only) as a background control. (TIF) [file pntd.0007687.s001.tif]

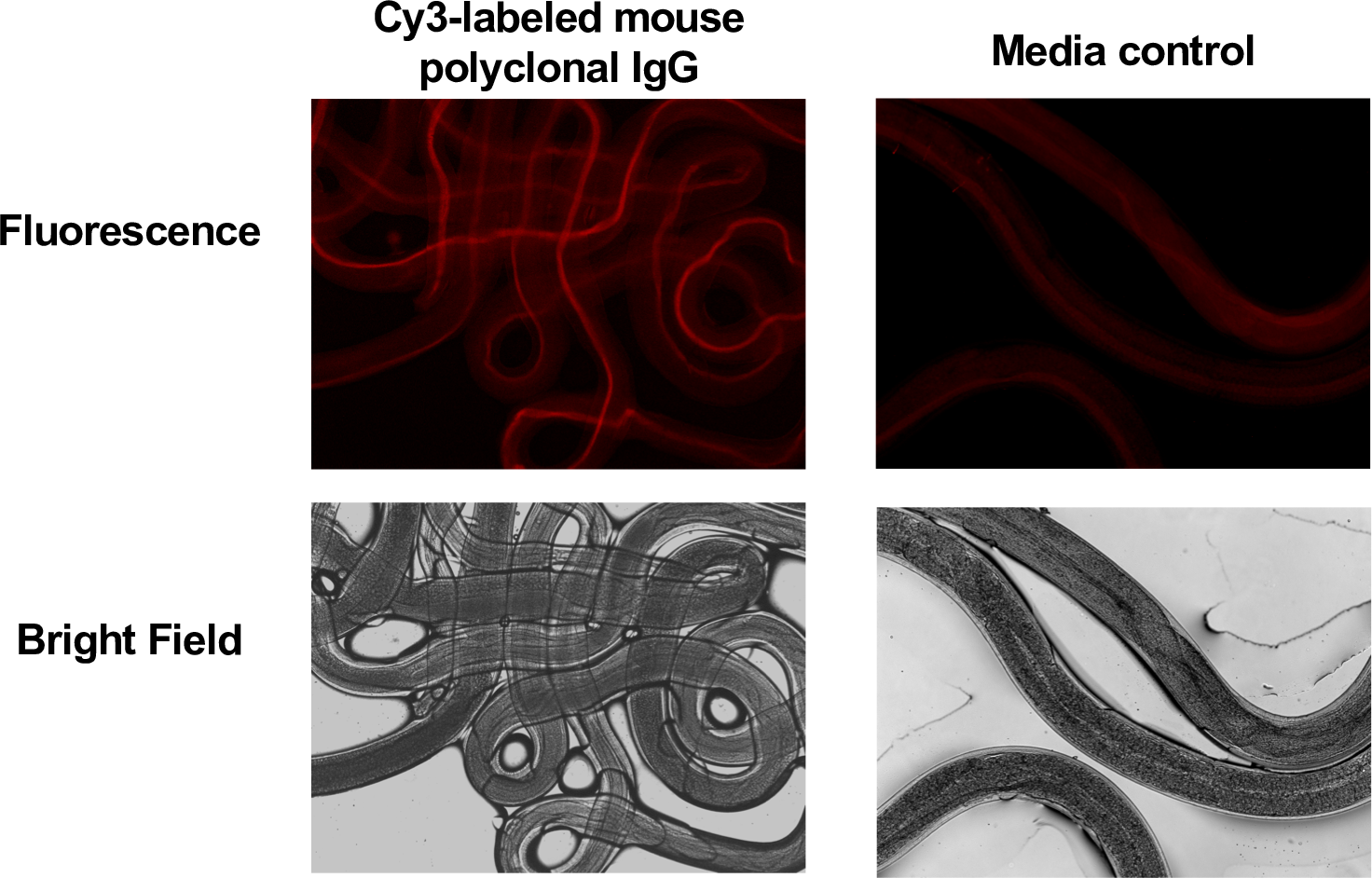

Supplement: S1 Fig — Adult worms were cultured for 24 hrs with Cy3-labeled mouse polyclonal IgG or in media alone. Ingested antibody is visible in the intestinal of the adult filaria. (TIF) [file pntd.0007687.s002.tif]

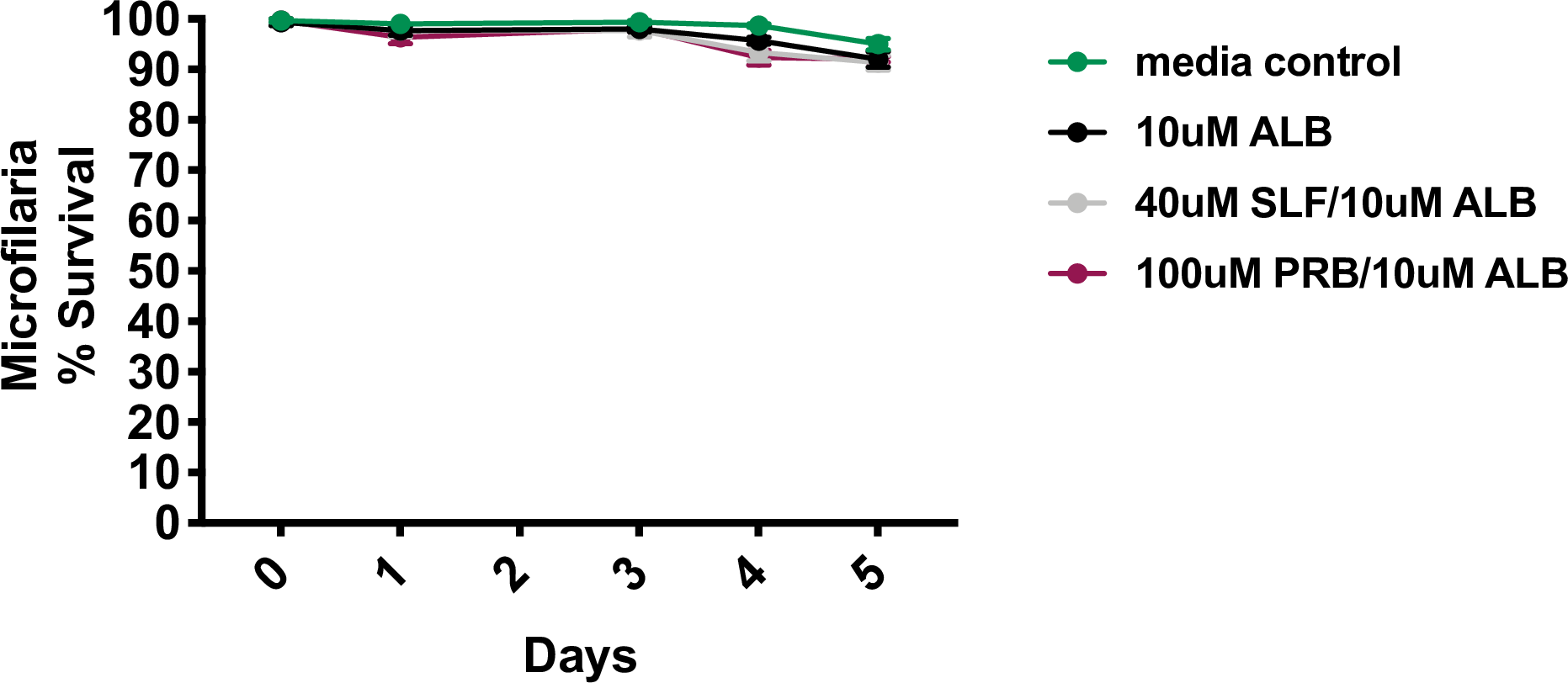

Supplement: S2 Fig — Percent survival of microfilariae incubated with 10 μM albendazole and 40 μM sulfinpyrazone (ns) and 100 μM probenecid (ns). Percent survival was calculated wells containing 2.0 x 10^6^ Mf similar to Fig 6. The generated AUC values were analyzed using an ordinary one-way ANOVA to determine significance followed by Tukey’s multiple comparison test. This experiment was only performed once. (TIF) [file pntd.0007687.s003.tif]
